# Visual cavity analysis in molecular simulations

**DOI:** 10.1186/1471-2105-14-S19-S4

**Published:** 2013-11-12

**Authors:** Julius Parulek, Cagatay Turkay, Nathalie Reuter, Ivan Viola

**Affiliations:** 1 Department of Informatics, University of Bergen, Norway; 2 CBU, University of Bergen, Norway; 3 Vienna University of Technology, Austria

## Abstract

Molecular surfaces provide a useful mean for analyzing interactions between biomolecules; such as identification and characterization of ligand binding sites to a host macromolecule. We present a novel technique, which extracts potential binding sites, represented by cavities, and characterize them by 3*D *graphs and by amino acids. The binding sites are extracted using an implicit function sampling and graph algorithms. We propose an advanced cavity exploration technique based on the graph parameters and associated amino acids. Additionally, we interactively visualize the graphs in the context of the molecular surface. We apply our method to the analysis of MD simulations of Proteinase 3, where we verify the previously described cavities and suggest a new potential cavity to be studied.

## Introduction

Molecular biology is studying biological phenomena on the highest magnification level where the life processes are carried out by interactions of molecular machinery. One key focus of this scientific branch is to study and determine the molecular structure, while another attention is given to its dynamics and interactions with the other molecules. The structure, or conformation, of a protein can for example be obtained through the crystallography and the interactions of the protein with its environment are modeled by means of Newtonian physics, involving potential energy, where induced forces modify the structural arrangement of the molecule. They are often referred to as molecular dynamics (MD) simulations. The outcome of the simulation is then stored as a sequence of transformations for each atom of the molecule or environment, denoted as trajectories.

The studied macromolecules such as proteins are typically analyzed for a binding site to act as a carrier of an important chemical substance. Alternatively, a small molecule is searched for that would change the conformation of a particular protein and by the structural change influence a certain chain of molecular interactions, called as pathways. For example in a pathway of a certain cancer types, one would like to change the conformation or to block the binding site of a participating protein to disable a successful execution of the pathway.

Typical questions raised by molecular biologist in their exploratory workflows are where is a suitable binding site, what are its chemical characteristics and how stable this binding site is over the simulated time. Typical carriers and binding sites are channels, pockets, and cavities on the molecular surface.

One way of channel and pocket detection and analysis is to perform the Monte Carlo sampling over the boundary of the macromolecule. Cavities can be identified and characterized by means of differential geometry on the molecular surface [1,2]. These techniques are mostly quantitative and non-visual.

Parallel to these approaches are analytical methodologies that utilize visualization of the molecular surface where the biologist assesses the molecular structure qualitatively and searches for potential binding sites. For this type of analysis it is very important that shape and depth cues are effectively communicated to the viewer [3].

**Figure 1:**
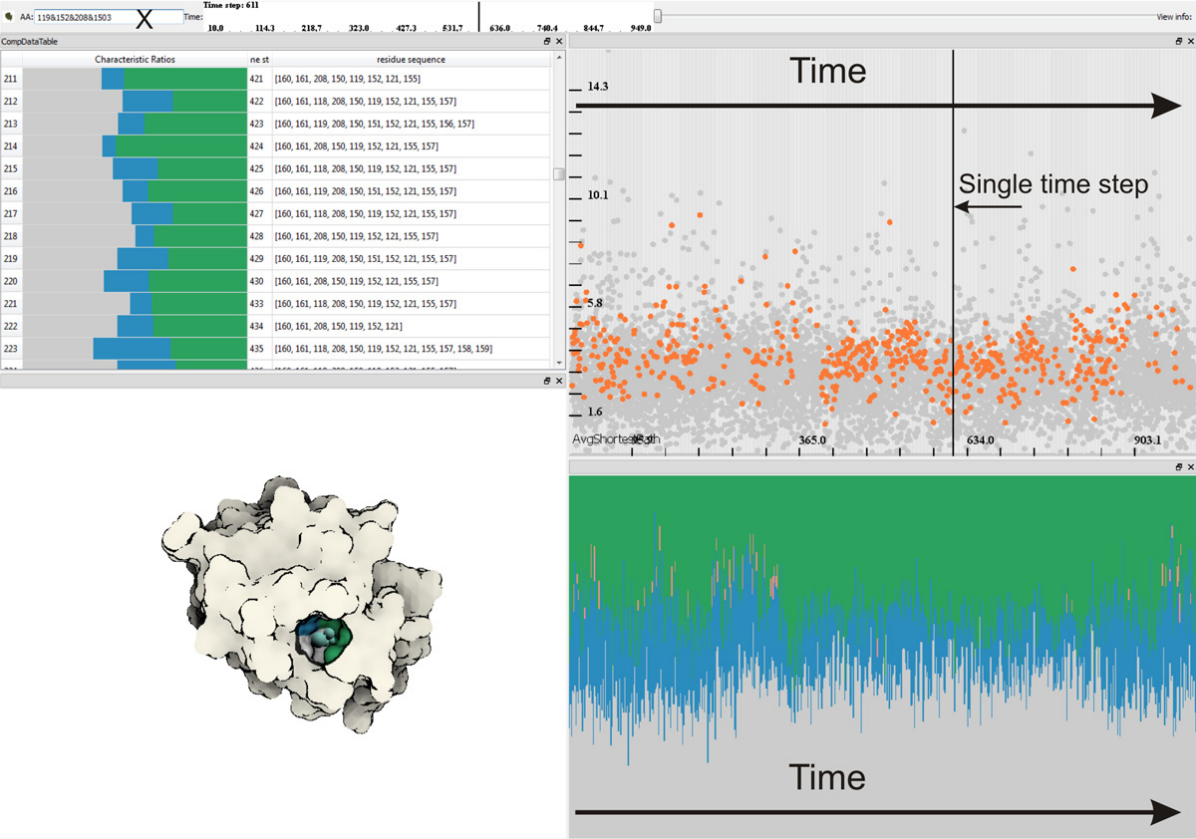
**An application screenshot**. Bottom-left: A 3*D *view shows a visualization of Proteinase 3 at time step 0. Top-left: An amino acids list view, where for each selected graph/cavity (a row), the cavity's amino acids are displayed. The color bar diagram represents a chemical property of the cavity with respect to hydrophobicity (gray), polarity (green), positive (blue) and negative (red) charges. Top-right: A temporal scatterplot, depicting an average graph size, can be used to select arbitrary graphs (selected graphs -- orange, non-selected -- gray), realized by mouse interaction or direct amino acids specification (X), which are then linked with the contextual 3*D *view. Bottom-right: A plot depicts chemical properties of cavities over the entire temporal domain.

We have identified the importance of the complementarity of these two approaches and propose a novel visual analytics framework for the cavity analysis. The cavity candidates are extracted automatically from the molecular structure for each timestep of the simulation. After the extraction process the user can visually analyze the cavity geometry, chemical properties and other important quantitative measures. The user can formulate a query for finding cavities that correspond to particular envisioned characteristics and by interacting with the temporal settings she can quickly get familiar with the binding site stability over time (Figure 1).

It is noteworthy that this work represents a natural continuation of our previous study [4], which focused mostly on the graph based cavity representation. Here we extend this technique by an improvement of the implicit function sampling and the 3*D *visualization, and also by characterization of the graph components by amino acid types.

## Related work

Our work can be regarded as related to two groups of techniques, namely implicit molecular representation, and cavity extraction.

### Implicit molecular representation

In order to model complex and dynamic geometric objects, implicit surfaces are a suitable mechanism. In the molecular visualization field, implicit representation has been used widely to smoothly model the bond transitions between single atoms. Blinn [5] used the set of techniques for the first time, which are today known as implicit modeling. In order to describe the electron density function of the atoms, he utilized an implicit function that sums up the contribution from the atoms:

(1) $$f\left(p\right)=T-\underset{i}{\sum }b_{i}e^{-a_{i}d_{i}^{2}},$$

with *d_i _*as the distance from **p **to the center of atom *i*, *b_i _*as the "blobbiness", *a_i _*as the radius of the atom, and *T *as a threshold for the electron density. In later studies, implicit surfaces that are constructed from skeleton points were introduced [6,7]. In general, these representations can be formulated as:

(2) $$f\left(p\right)=T-\underset{i}{\sum }m_{i}f_{i}\left(p\right),$$

where *m_i _*is a weight factor and *f_i _*is a density distribution function that is decreasing. Shestyuk [8] presented a comparative analysis on how different distribution functions can be applied. The performance of the kernel evaluation in the rendering process was improved by GPU implementations [9], which were later used for fast visualization of molecular surfaces [10,11]. The above approaches that use the summation of atom contributions can be considered to be relatively fast and thus widely used. However, these approaches do not completely consider the solvent that is usually represented as a sphere with radius *R*. The consideration of the solvent, on the other hand, can lead to valuable findings that can lead to potential binding sites.

Pasko et al. [12] combined different implicit model forms to propose a generalized implicit surface representation. The implicit object representation is denoted as a function that involves the following inequality:

(3) f(p)≥0,

where **p **= (*x*_1_, *x*_2_, *x*_3_) ∈ *E*^3 ^and *f *is an implicit surface function (or implicit function). *f *classifies the space into two half-spaces: *f*(**p**) *>*0 and *f*(**p**) *<*0. The above classification is also valid for Eqs. 1 and 2.

There are a number of methods to represent molecular surfaces. A common approach is to represent atoms as spheres with radii that amounts to the van der Waals forces (vdW surface) [13]. The implicit function for the van der Waals that follows Eq. 3 is defined as: $f\left(p\right)=\cup_{i}\left(r_{i}-d_{i}\right)$, where *r_i _*is the van der Waals radii. By extending the surface with a solvent radius, one obtains a solvent accessible surface: $f\left(p\right)=\cup_{i}\left(\left(r_{i}+R\right)-d_{i}\right)$.

In the cavity exploration area, the most common representation is the solvent excluded surface(*SES*) [14]. Recently, Lindow et al. [15] and Krone et al. [16] proposed GPU implementation of the *SES *representation. Although they achieved a high rendering performance, their models are solely applicable to rendering related tasks. Our cavity detection method, introduced in this work, requires that the molecule is defined as an implicit surface.

Parulek and Viola [17] introduced a functional representation for the modeling and the visualization of the *SES *representation. In their method, the molecular surface is represented as a combination of basic *CSG *operators and they define a distance based implicit function. Our function sampling procedure uses this representation as a basis. Further details are in the Visualization section.

One method to visualize implicit molecular models is to construct a mesh representation and render the mesh as a set of patches [18]. However, in the case of complex molecules the resulting meshes can consist of millions of triangles, which creates a challenge to generate detailed iso-surfaces. As a result, direct visualization techniques such as ray-casting have been introduced recently.

One subclass of implicit surfaces are represented by distance based functions. Effective visualization of such objects was proposed by Hart [19]. Since, essentially, the distance measure for an implicit function can be approximated by the first Newton iteration of the function:

(4) $$f_{dist}\left(p\right)\approx \frac{f\left(p\right)}{\left|\nabla F\left(p\right)\right|};$$

we also adopted Hart's technique for rendering.

### Analysis of protein cavities

Since the empty spaces on protein surfaces provide valuable information, they have been investigated widely in the literature. Many methods utilize the analytical description of the *SES *[20]. For instance, Voss and Gerstein [21] introduced a web-based cavity analysis tool that apply two separate probes to calculate the solvent volume to search for potential cavities and channels.

There are also several tunnel exploration methods. In general, these methods require the specification of an initial point in the empty space within the protein. The method tries to reach the exterior by following tunnel-like cavities and fills the space with geometric structures as it progresses. These methods also provide information related to the pathway around the exit area to describe the cavities. The method HOLE [22], uses a similar strategy, where the user defines the initial location and orientation of a pore within the molecule. The specification of the initial parameters have been automated by Coleman and Sharp [1], where their algorithm is also capable of determining arbitrarily shaped tunnels. Voronoi diagrams have been used to discover molecular channels and pores in CAVER [23] and MOLE [24]. Recently, Voronoi diagram of spheres showed its potential to extract significant paths from the molecules [25]. Random rays are generated at Voronoi vertices in order to remove them outside the molecule. Although the use of ray casting to determine cavities is similar to our method, we utilize an implicit function sampling rather than Voronoi vertices. Our method puts more emphasis on the molecular surface.

Pore features are utilized to determine channels in an iterative and heuristic algorithm in Pore-walker [26]. Within the context of tunnel extraction methods, our approach can be described as a combination of stochastic methods due to use of function sampling, and Voronoi diagrams due to use of graph analysis.

Molecular pockets and cavities have also been subject to many studies. CAST uses computational geometry together with alpha shape theory in order to extract cavities [27]. Till and Ullmann use a Monte Carlo algorithm while sampling a protein surface over a 3D grid [2]. Although the use of randomly sampled points to calculate cavities is similar to our method, we directly use the sample points to estimate the cavities rather than using a 3*D *regular grid. Moreover, our approach also includes the use of interactive visual analysis to investigate the resulting cavities.

A grid-based approach that also considers molecular dynamics is utilized in identifying internal cavities and tunnels [28]. Similarly, Krone et al. [11] introduce a technique to track the evolution of the cavity in dynamic cases. In our work, we do not focus on tracking cavities. Instead, we present a set of potential cavities for each time step, where the user has the functionality to explore this set of cavities through linked views and interaction.

## Method overview

To represent molecular surfaces by an implicit function *f*(**p**), we employ the approach introduced by Parulek and Viola [17]. Nevertheless, one can use a kernel based approach (Eq. 2), and as well as vdW or SAS, which both can be easily expressed as implicit functions. The implicit function is positive inside the molecule and negative outside, and it is possible to estimate the minimum distance of a sample point from the surface. The distance can be computed by the application of Newton's formula, (Eq. 4).

**Figure 2:**
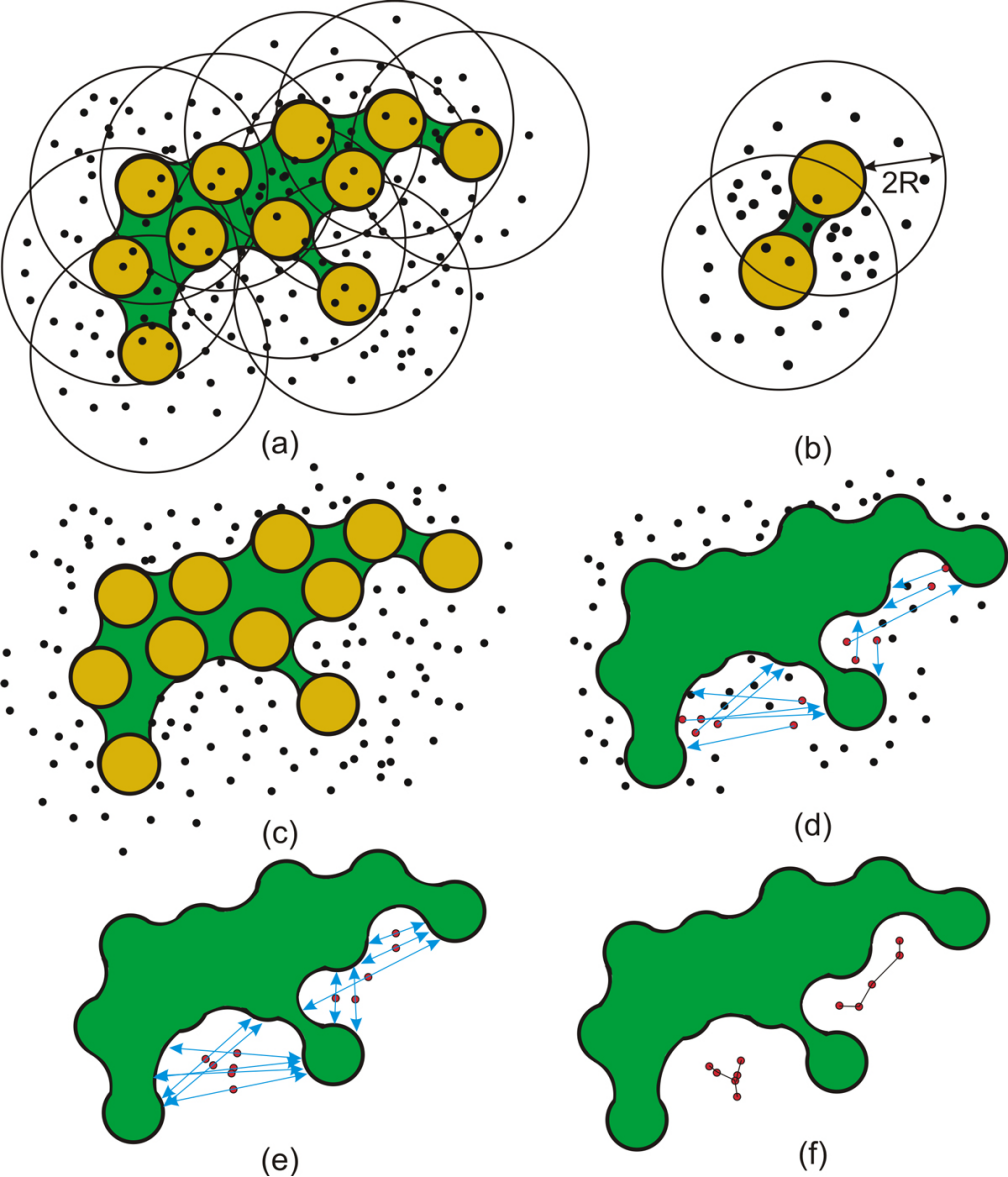
**The pipeline for detection of cavity samples**. a) A set of random samples is seeded in the space delimited by the radius 2*R *from the van der Waals spheres (yellow circles). b) Generation of sample points for two atoms. Note that more sample points are obtained in the intersection of both enlarged spheres. c) The samples **p **that lie inside the molecule (*f*(**p**) *≥ *0), are excluded. d) Detection of cavity samples is performed by means of shooting the ray (blue) along the gradient direction evaluated at all the samples. Those samples that hit the iso-surface (red) are labeled as potential cavity samples. Here only the rays that hit the iso-surface are rendered. e) The new sample position is computed, which is defined as the middle point between two points obtained by ray iso-surface intersection. f) The resulting graph components after the application of connected component and minimum spanning tree analysis.

Similarly to our former study [4], we compute an independent set of graphs, $G^{t}=\left\{G_{1}^{t}\cup \ldots \cup G_{m}^{t}\right\}$, representing *m *cavities of MD simulation in time step *t *(Figure 2). Here we improve the positioning of sample points forming the graphs. These samples are generated with respect to atom centers within radius [*r_i_*, *r_i _*+ 2*R*] from each atom, i.e., within the influence of the atom. Moreover, for each cavity graph $G_{i}^{t}$, we compute graph parameters, e.g., the average shortest path, and amino acids that compose the molecular surface near the graph. The user is provided with the system of linked views allowing her to select individual graphs according to the graph parameters and as well as by direct amino acids specification.

### Cavity graphs

In the first stage, we sample the implicit functions by a set of random points, *S *= {**p**_1_, ..., **p***_n_*}, which densely cover the function domain (Figure 2a). One of the important issues related to cavity extraction from the molecular implicit function is how to prefer regions with higher surface complexity. This is due to the fact that the occurrence of the cavities is directly related to the surface complexity. In another words, we should emphasize surface regions with a higher curvature variation. Fortunately, this is highly correlated with respect to the density of atoms in that region, since the function evaluation employs the closest atoms only, i.e., within distance *r_i _*+ 2*R *from the *i*-th atom. Therefore, the sampling can be performed by generating an equal number of sample points for each atom, which will naturally create more sample points in regions with more atoms, i.e., in regions with higher surface variations (Figure 2b). We perform the sampling for every time step of the MD simulation, where the positions of sampling points remain almost the same for all the time steps, i.e.; we slightly adjust the position with respect to the molecular bounding box in a particular time step.

The sampling process evaluates the implicit function *f *at every sample position; i.e., we obtain a set of function values *F^t ^*= {*f*(**p**_1_), ..., *f*(**p***_n_*)} for time step *t*, where *n *represents the number of samples. With respect to the property of implicit functions that classifies points between internal and external ones, we can easily filter out samples *S*_0 _⊆ *S *that lie inside the protein, *S*_0 _= {**p***|f*(**p**) *≤ *0; **p **∈ *S*} (Figure 2c), which do not belong to any cavity.

Essentially, *S*_0 _contains a set of sample points lying in a close vicinity of the surface, up to a distance of maximum 2*R *from the molecular surface, which is clear from the sample point definition.

As a next step, we perform a cavity based analysis, which classifies the samples into potential cavity samples. Note that there is no exact cavity definition with respect to any of aforementioned molecular surface definitions, i.e., van der Walls spheres, solvent accessible surface, solvent excluded surface, blobby models, etc. Nevertheless, there are at least some hints on how the cavity can be described. In our work we follow the specification by Cheng and Shi [29], which describes a cavity as a connected and concave surface patch that might open up to the outside via a narrow mouth. This property allows to define the cavity through opposite facing surfaces. This condition is verified at each sample by a ray that is cast along the normal direction beginning at the sample. In a case that the ray hits the surface, the sample is classified as a potential cavity sample [4] (Figure 2d). Thus only the samples that lie between two opposite facing surfaces are labeled as a potential cavity. Although this excludes more shallow regions, it was still preferred and recommended by our collaborators from bioinformatics. On the other hand, the ray-casting method can be performed in a more robust way, such as for instance producing multiple rays in various directions. Nevertheless, casting just a single ray is a very fast method and, when taking into account the large number of employed samples, it also filter out many false positives in the set *S*_0_. Afterwards, we adjust the sample position to lie in the middle of two opposite facing surfaces (Figure 2e).

The number of points (samples) that are seeded to the spatial domain depends primarily on the size of the molecule: for instance for Proteinase 3 (3346 atoms), used in our use case, we employed 16 samples per atom, i.e, 3346 *× *16 = 53536 of sample points. The number is significantly lower than in approaches that employ regular grid discretization, e.g., 256^3 ^stands for 16777216 sample points. In practice the number of sample points is evaluated with respect to acquired cavities, i.e., we gradually increase the number of samples, and when after a certain number of samples the amount of extracted cavities does not change dramatically, this number defines the amount of required samples.

In the next stage, our goal is to form a graph that defines the relations between the cavity samples. First, we perform visibility tests between all pairs of sample points. This generates an undirected graph *G*, where nodes are the sample points and edges are mutually visible samples. Secondly, we perform the connected component analysis, which results into the set of *m *independent subgraphs *G *= {*G*_1 _∪ ... ∪ *G_m_*}. Thirdly, we apply a minimum spanning tree algorithm [30] to each component *Gi *to build its central skeleton (Figure 2f).

For more details on our graph extraction technique, we refer readers to our previous study [4].

### Visualization

The rendering of implicit surfaces representing molecules by a single distance based function was introduced by Parulek and Viola [17]. In our previous study, we improved the proposed pipeline by utilizing spherical impostors representing an area of the atom influence [4].

**Figure 3:**
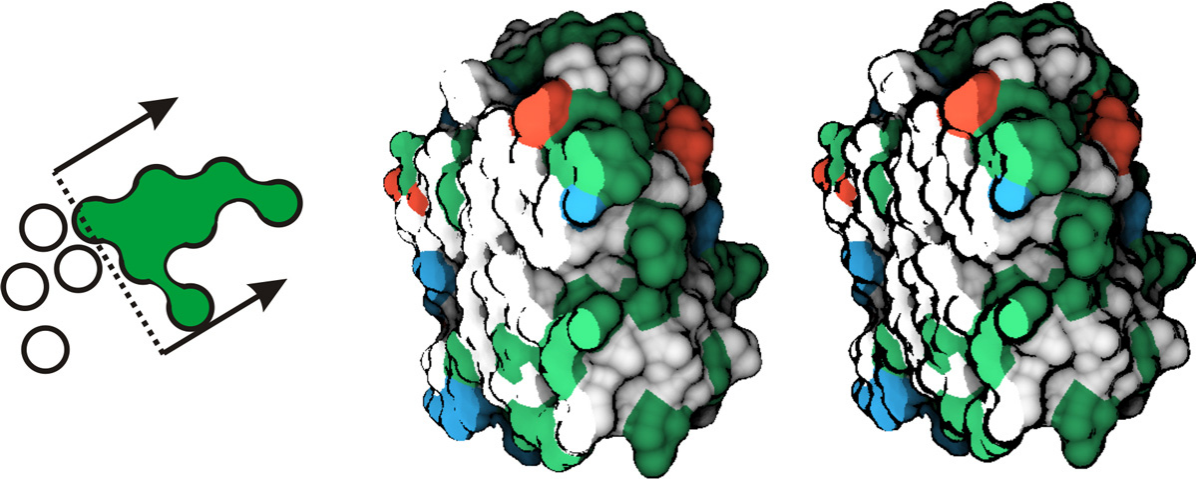
**Implicit clipping plane and contour enhancement**. Left: The implicit function evaluates the molecular surface (green). It takes into account only atoms that intersect the plane or lie in the half-space defined by the plane (arrows). Middle: Proteinase 3 is colored according to the amino acids, while for the clipped surface the flat shading model is employed. Right: An example of changing the width of contours.

To ease the shape perception, we farther improve the surface rendering by contour enhancement. In the literature, there are several papers on contour enhancement techniques [31]. The simplest one employs the angle between the surface normal and the viewing direction. The disadvantage is that flat boundary regions that have similar gradients may become a part of the contour as well. Therefore we turn to curvature-based techniques, which can suppress contours in low-curvature regions. On the other hand, those techniques are usually computationally demanding. Therefore, we adopt a technique introduced by Bruckner and Gröller [32], which approximates the view-dependent curvature by evaluation of two consequential gradients along the viewing ray. Moreover, it easily allows to change contour thickness (Figure 3 middle and right).

The surface color is determined by the amino acid type. The amino acids are the basic building compounds of molecules, and also provide a deeper relation for biologists with our cavity analysis. We classify the amino acids into four categories, according to the classical amino acid Venn diagram [33]. The four categories of amino acids are hydrophobic (white), negatively charged (red), positively charged (blue) and polar ones (green). The final surface color is determined by the closest amino acid with respect to the surface point.

To allow for molecule exploration, we include a clipping plane interaction, which we refer to as an implicit clipping plane (ICP). The ICP clips away the atoms from the implicit surface. This enables us to study even occluded cavities located inside the molecule. Here we exploit the fact that the implicit function is constructed on the fly during the ray-casting. The ICP neglects those atoms that lie in front of the clipping plane (Figure 3 left). The reason for using such a clipping plane is to preserve the molecular surface in the close vicinity of the plane. Users can either link the plane normal with the viewing direction, or adjust the plane orientation interactively. Additionally, when the implicit clipping plane is activated, the diffuse shading model is evaluated just for the surface area that is not clipped. This enables us to distinguish between the clipped surface and the original one. For the clipped surface points we utilize just constant colors derived from the amino acid type (Figure 3 middle and right).

**Figure 4:**
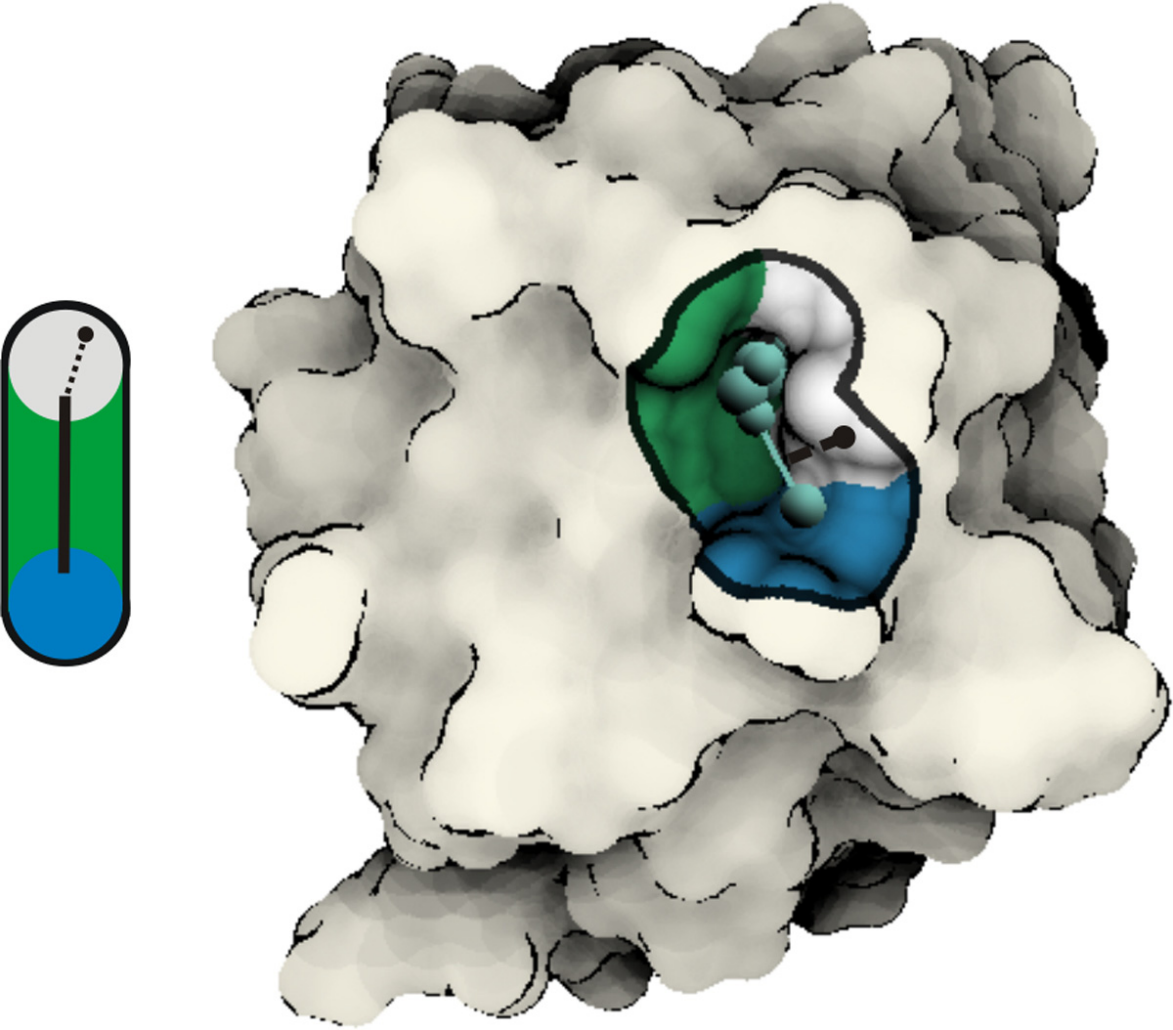
**Visualization of graph components**. Left: The iso-surface point (the black circle), obtained during ray-casting, is evaluated against the distance (the dashed line) to the graph component (the black line). Right: An example of graph component visualization in the context of the molecule. When a graph component is shown, the coloring is applied only to points that lie within distance *D_g _*= 3*R *from the graph. We employ flat shading for surface points lying beyond *D_g_*. The boundary of *D_g _*is shown as a black contour on the surface. The graph component is displayed using line segments (edges) and spheres (nodes).

To depict the graph components, we use basic geometrical primitives, i.e., spheres and line segments. The radii of spheres are defined by the sample distance from the molecular surface [4]. The edges represent the minimum spanning tree of each graph. Our system allows to select and visualize a group of graphs for each time step separately. We visualize the graph components in the focus and context style. The focus, the molecular surface close to the selected graph component, is colored using the amino acid type, whereas the context, the molecular surface farther away from the selected graph component, is shaded constantly (Figure 4).

## Graph attributes

The cavity extraction procedure generates tens of graphs per time step over a simulation containing thousands of time steps. Therefore, direct integration of all the graph components into the visualization can easily produce results that are cluttered and difficult to interpret. In our former study [4], we introduced an interactive system that allows performing visual selection of the graph components to steer the focus of the cavity analysis. To ease the graph exploration, we compute a set of basic graph measures: the longest path between any two nodes, the average length of the shortest paths between pairs of nodes (*avgP*), and the average of the degree of all the nodes. In our examples we employ *avgP *for selections, which essentially represents the overall cavity size.

**Figure 5:**
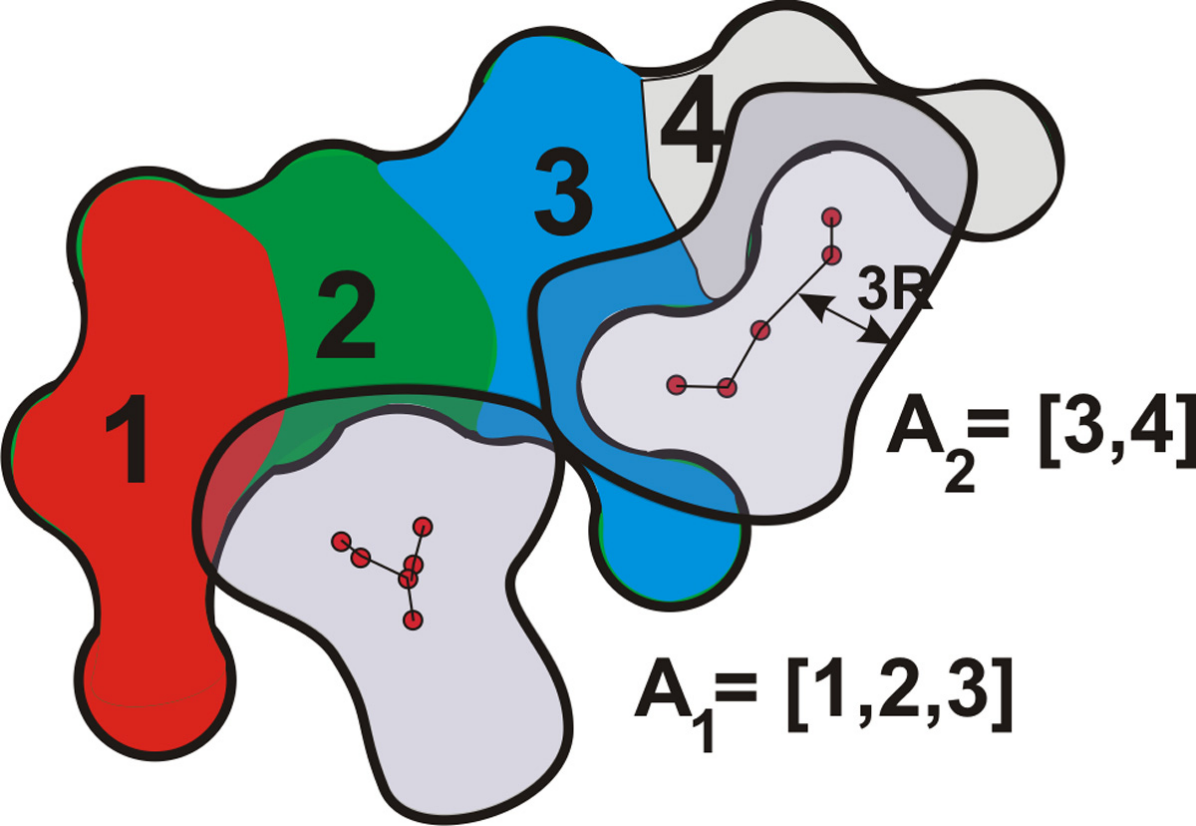
**An illustration of the assignment of amino acids to the graph components**. We turn the cavity skeleton into a distance object, bounded by the distance *D_g _*= 3*R*, and perform an intersection with the molecular implicit function. We mark those atoms/amino acids that form the molecular surface.

Additionally, we compute amino acids (*A_i _*= {*a*_1_, ..., *a_k_*}) that compose the molecular surface near the cavity graph *G_i_*. Here, we employ the geometrical distance *D_g _*= 3*R *from the cavity graph, i.e., if there is an intersection between the molecular surface and the graph component (Figure 5), we assign the amino acids composing the surface to the graph. The assignment of amino acids is illustrated in Figure 5.

By utilizing the properties of the amino acids *A_i _*(assigned to the cavity *G_i_*), we compute a profile of the cavity *G_i_*. We build this profile by utilizing a categorization of amino acids based on their chemical properties [33], i.e., the same as we employ for the surface colors. In order to build the profile of a cavity according to these categories, we iterate through the atoms that form the molecular surface near the cavity graph (Figure 5). We mark each atom according to the type of the amino acid it belongs to, e.g., if an atom is a part of a polar amino acid, it is considered to be polar. After all the atoms are marked, we count the number of atoms and compute the ratios for each category. We use these ratios to visually represent the profile of a cavity, where each category is mapped to a color: gray for hydrophobic, green for polar, blue for positively charged, and red for negatively charged amino acids.

## Interactive analysis of graph components

The computation of the graphs and their attributes results in heterogeneous data related to the simulation. At this stage of the analysis, we have three different types of data involved in the visualization: i) the raw simulation data ii) the graph components data iii) the amino acids data. In order to analyze these heterogeneous data, we make use of a coordinated multiple view setup that employs interactive visual analysis (IVA) methods. Our setup employs linked views, where each type of view can handle different parts of the data. Firstly, to visualize the raw simulation data, we make use of the 3D visualization method previously discussed (Figure 1 bottom-left). Secondly, we utilize a scatterplot that visualizes a selected graph attribute (*y*-axis) over time (*x*-axis), where each dot represents a unique graph component (Figure 1 top-right). Finally, two separate views show the data related to the amino acids. One view visualizes the chemical properties of cavities (cavity profiles) over time (Figure 1 bottom-right) and another view lists the selected cavities and their amino acids ordered by time (Figure 1 top-left).

These different views are linked using an interaction method called linking & brushing. This method enables the user to interactively make selections (also called brushes) in one view and observe what structure the same selection corresponds to in the other views. In order to visually express the selection in the views, we make use of two methods. In the first method, we highlight the selected data in the context of the whole data. Example of this method could be seen in the graph attribute scatterplot, where the selected graphs are highlighted by orange color and the rest of the graphs, the unselected ones, are displayed in gray (Figure 1 top-right). The second method displays only the selected information. An example of this method is the cavity profile view, where only the profiles of the selected graphs are shown (Figure 1 bottom-right).

In our system there are two different ways to select graph components. The user can either interactively select (brush) the graphs through the graph attribute scatterplot (Figure 1 top-right) or specify the amino acids through textual queries. Additionally, different selections can be combined via the basic Boolean operators (*AND*, *OR *and *NOT*), which lead to more complex queries.

**Figure 6:**
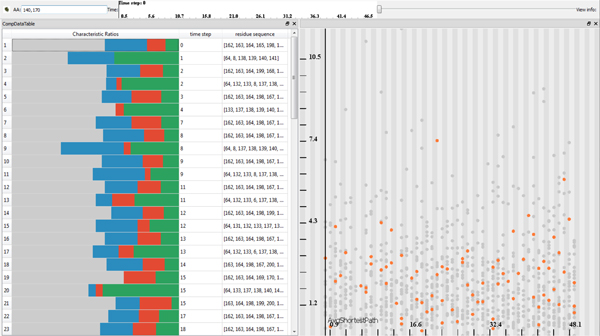
**An amino acids list view and a temporal scatterplot showing *avgP *over time**. Here we specify those cavity graphs that are formed either by amino acid 140 or by 170, i.e., the query equals 140 ∨ 170. Left: The leftmost column depicts the characterization of cavities with respect to the occurrences of amino acid types: hydrophobicity (gray), polarity (green), positively (blue) and negatively (red) charged. The middle column shows the time step the cavity occurs at. The rightmost column shows all the amino acids forming the cavity. Right: In the accompanied scatterplot we see the distribution of the selected cavities over time with respect to its "size", *avgP*.

One important point to mention is that all the views are updated automatically whenever a selection is made. For example, in the amino acid list view it is possible to select cavity graphs through a direct specification of amino acids that are of interest, and the other views display the selection immediately. Through this view, the user composes textual queries that include *AND *= & and *OR *= ∨ operations. In Figure 6 we specify two amino acids 140 ∨ 170, which selects graphs *g *= {*G_i_|*140 ∈ *A_i _*∨ 170 ∈ *A_i_*; *G_i _*∈ *G*}. In the accompanied scatter-plot we can observe the distribution of these graphs, *g*, over time. Additionally, it is possible to combine queries by specifying the intervals of amino acids, e.g., the query (120 *− *140)&(180 *− *190)&173 represents all cavities that contain at least three amino acids *a_i_*, *a_j_*, *a_k_*, such that *a_i _*∈ [120, 140], *a_j _*∈ [180, 190] and *a_k _*= 173.

## Implementation and performance

We implemented the entire system in Python programming language, where most of the rendering and computations run on the GPU (CUDA and GLSL). The performance measurements are done on a workstation equipped with two 2 GHz processors and 12.0 GB RAM, and with a GPU NVIDIA GeForce GTX 680. The 3*D *cavity visualization in the context of the molecular surface is performed on the fly (GLSL for sphere billboarding and CUDA for ray-casting). The molecular visualization can be performed even without the cavity segmentation, since the ray-casting pipeline is independent from the graph analysis. Prior to 3*D *rendering and cavity segmentation, the only auxiliary structure that needs to be computed is the GPU representation of atoms.

We utilize a simple and straightforward approach that is based on an uniform spatial subdivision. This has been already utilized by the broad molecular visualization community [15,16]. The atoms are sorted into cubic voxels with a lateral length of 2*radius_max _*+ 2*R_max_*, where *radius_max _*represents the maximum (van der Waals) radius of all the included atoms and *R_max _*represents the maximal allowed solvent radius. Then, in order to find the closest atoms to a given point, it is required to visit 3 *× *3 *× *3 neighboring voxels. Thus, for a given time-step, we need to send to the GPU only the atom centers and their radii, and the grid of voxels. Such a grid of voxels is computed and stored automatically when the user selects a particular time step either to visualize or analyze, which has not been processed before.

In the process of cavity segmentation all the samples are precomputed for the entire simulation, where the user has the possibility of resampling a particular time-steps if desired. All the samples are evaluated in parallel, time-step wise, using CUDA. For instance, evaluating and segmenting 50*K *samples for 1000 time steps takes around 20 minutes. After the cavity samples have been segmented, the user can initialize the computation of graph components. The generation of graphs takes around 10 minutes for 1000 time steps, for the previous example. The process of assigning amino acids to the generated graphs is automatically executed after the graphs have been formed. This takes approximately another 10 minutes. After these pre-processing steps are over, the system operates at interactive rates. It is important to mention that, even when performing complex queries constructed through our selection mechanism, the system gives an immediate response.

## Use case: analysis of Proteinase 3

Proteinase 3 (PR3) belongs to the family of serine proteases, cleaving proteins via specific hydrolysis of peptide bonds. It is an enzyme involved in inflammation, where in a number of chronic inflammatory diseases, e.g., Wegener granulomatosis and vasculitis, PR3 has a deleterious effect. Therefore, PR3 is a drug target. To design drugs for PR3, we need first to understand of how ligands bind to it, which is conditioned by a better characterization of the binding sites. This allows the development of drug candidates with higher affinity to PR3 than its endogenous targets [34].

The search for new drugs often relies on knowledge of the three-dimensional structure of the enzyme involved, and in particular of the cavities on its surface. The drug candidate efficiency is dependent on a strong interaction with the enzyme. The strong interaction can be achieved by binding into a cavity. Nevertheless, all molecules are dynamic and the structural changes they undergo impact their function. This is also valid for the dynamics of cavities. Thus our goal is to provide dynamic picture of the relevant cavities over the simulation time.

The analysis starts with importing the *PDB *and *DCD *files for PR3. The Protein Data Bank *PDB *file format is the most common format for atomic cartesian coordinates and other relevant information (e.g., atom types, amino acid types, sequence numbers). The *DCD *file format is commonly used for MD simulation trajectories, and is the output format of MD engines, such as CHARMM [35] or NAMD [36]. For demonstrational purposes, we limit the number of time step analyzed to 1000.

After loading the data, the user can already visualize the molecular surface in the 3*D *view. In the context menu that is available in the application, the user can select multiple commands that run the sample and the graph components generation. Here, one can decide to execute all the computations, i.e., samples evaluation, graph creation and amino acids computation, at once for the entire simulation or for each time-step individually.

Our framework computes automatically the number of occurrences of amino acids with respect to the graphs. Using this information, one can easily find the graphs/cavities, which refer to the most present amino acids in the MD simulation. Moreover, through *AND *and *OR *operations and the linked 3*D *view, one can verify whether those amino acids belong to the same cavity.

Another possibility is to verify a priori knowledge of the cavity that is formed by specific amino acids. Here the user can specify the corresponding amino acids queries by the *AND *operation, or by *OR *operation to see whether the occurrence of the selected graph components in the accompanied temporal scatter-plot has changed.

### Benchmarking against known binding sites

Here we firstly show how to perform validation of existing binding sites discoveries. Hajjar et al. [34] evaluated a binding site that had been early characterized as containing an isoleucine (Ile171) and an aspartic acid (Asp190). The characterization originated from visualization of the X-ray structure of Proteinase 3. Using MD simulations of Proteinase 3 with many different ligands docked in the binding site, Hajjar and coworkers showed that Ile171 and Asp190 did not play any significant role in the interactions with the ligands. Instead Ser176 and Val193, as well as possibly Ser191 were interacting with most of their ligands. Additionally, there might be another cavity formed by, among others, Asp190 and Ile1.

It is important to mention that these results were obtained by a series of MD simulations, where each simulation represents another ligand bound to Pr3. The analysis consisted in measuring the occurrences of contacts between the ligand and any amino acid of Proteinase 3. Here we show that, with our visual analysis framework, we can directly evaluate some of these binding sites by analyzing just a single MD simulation. Moreover, with our method, the analyst gets an overview of the existing cavities, characterized in terms of size and chemical properties.

**Figure 7:**
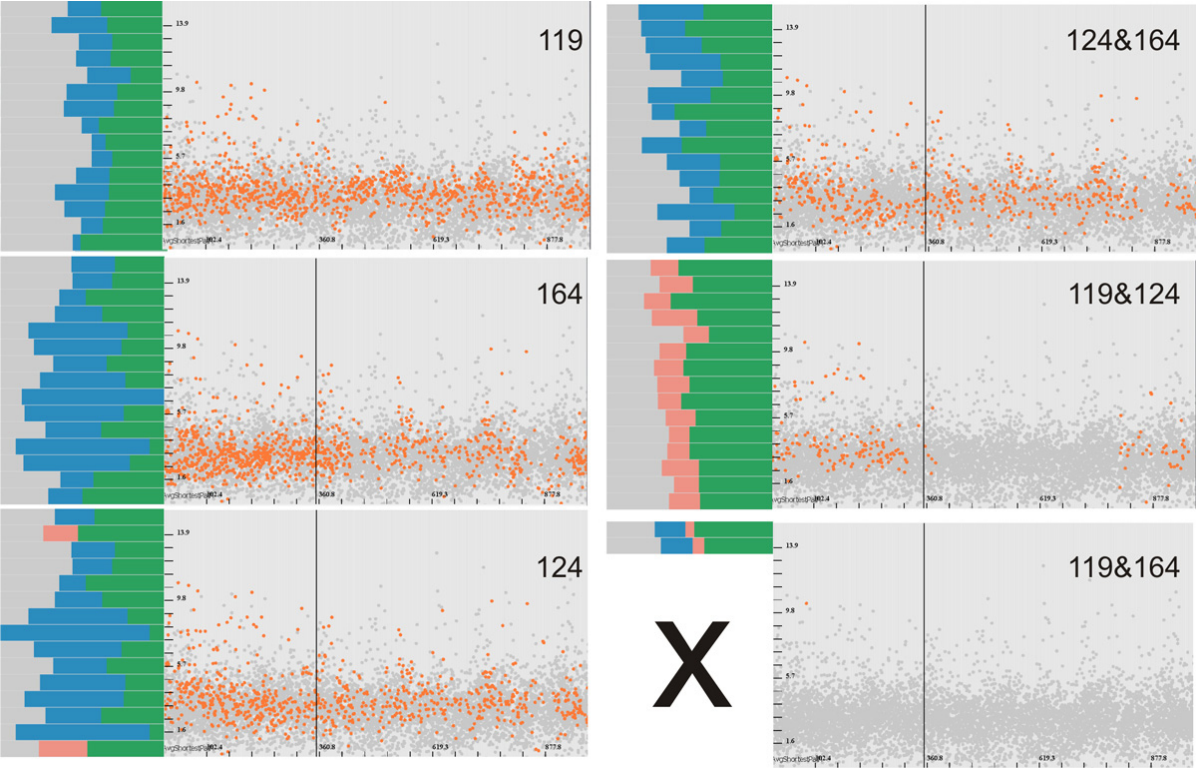
**Evaluation of existing cavities**. We sequentially perform selections of cavities based on pairs of amino acids. A typical cavity for each selection is shown in the top-left corner and marked by the green circle in the temporal scatterplot. Left: We can see that cavities formed by Ile171 and Asp190 are quite correlated with respect to the content of negatively charged amino acids. The same holds for amino acids Ile1 and Asp190, but only for graphs containing both, i.e., 1&190. Right: We found that there is a higher number of cavities formed either by Ser176 or by Val193, 176*v*191, than by both of them, 176&191. Note that the hydrophobicity of all the cavities is much smaller than in the cavities formed in the left side. We might see similar cavity occurrences when selecting cavities formed by Ser173 or by Val193 (176 ∨ 193) while cavities are more hydrophobic. Importantly, in the simulation section, we do not find any cavity when querying both Ser173 and Val193 (176&193).

**Figure 8:**
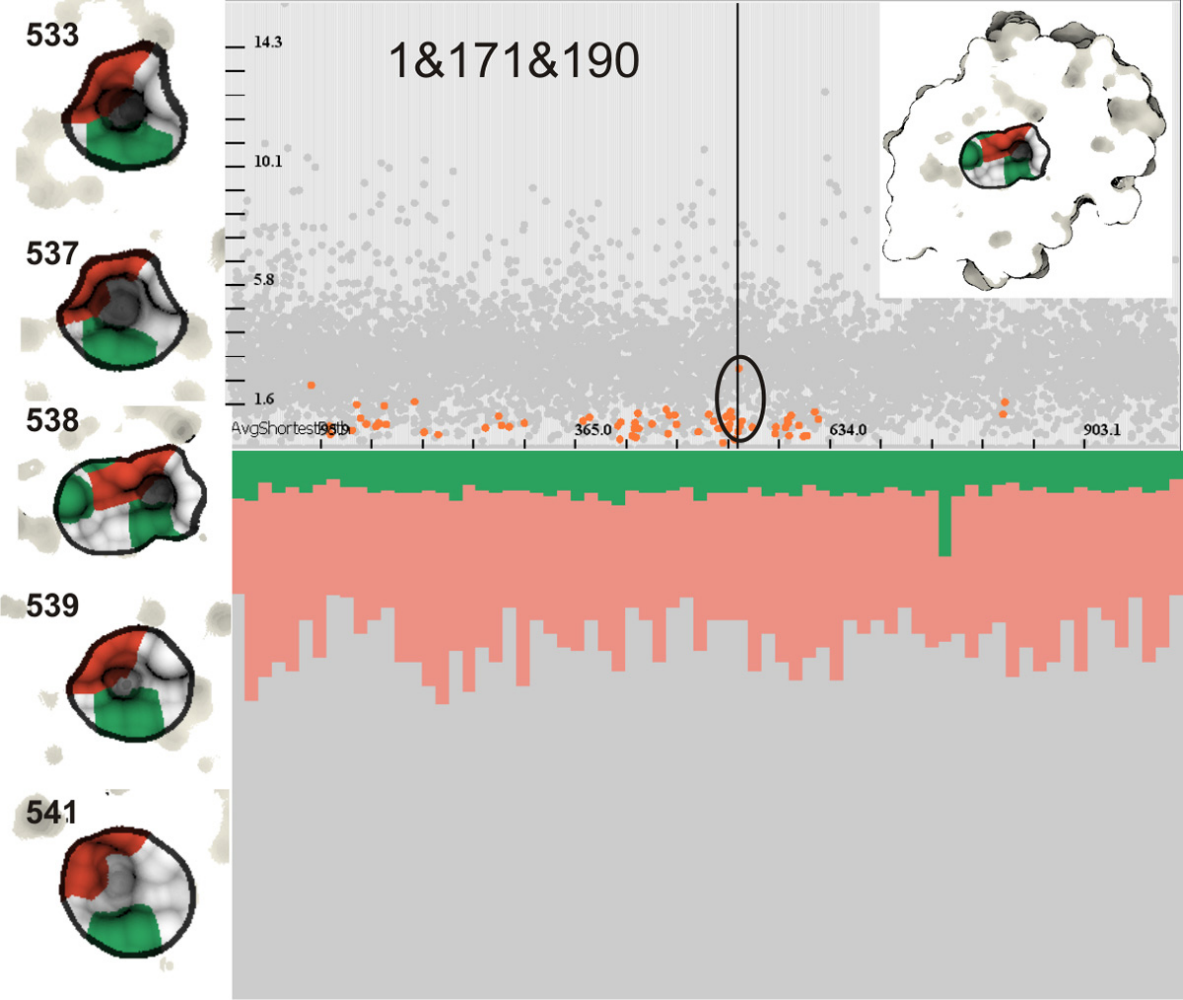
**A cavity formed by Ile1, Ile171 and Asp190 (1&171&190)**. Left: An illustration of of the same cavity in different time steps (533,537,538,539 and 541) marked by the black circle in the temporal scatterplot. We performed specification of 1&171&190 as a continuation of the analysis started in Figure 7, which shows that the cavity might be formed by all three amino acids. The cavity is located deep inside PR3 and we have to use the ICP to show it. Bottom-right: It is easy to see that the chemical properties of the cavity are very stable over entire simulation, where hydrophobicity prevails over polarity.

We create a system of views similar to Figure 1, where we analyze the first 1000 structures resulting from 1 nanosecond-long MD simulation of PR3 with a peptide ligand. The analysis is done solely on the protein PR3 to demonstrate the potential of our approach. After the computation of all the graph components, we perform different combination of *OR *and *AND *operations applied on the amino acids that we would like to evaluate. Firstly, we have a look at their distribution in form of cavity graphs over time and in the amino acid list view (Figure 7). For instance, when we specify in the amino acid list view 171&190 (both Ile171 and Asp190), we see in the linked views (Figure 7 top-left) that the cavity disappears in the middle of the simulation. This shows that, even though it exists in the X-ray structure (which is also used as a starting point for the MD simulations), it quickly disappears to reveal another cavity formed by other amino acids. This reveals that the other cavity is constituted of amino acids other than Ile171 and Asp190, namely Ser176, Ser191 and Val193. In what follows we will use only amino acid numbers to be consistent with the textual queries. Before we evaluate other amino acids, we firstly perform a visual correlation between cavities formed by 171&190 and by 1&190. In order to do that, we execute the query 1&171&190 (Figure 8) and see that it represents the same cavity defined in Figure 7-left. Moreover, this cavity is located deeply inside PR3 (Figure 8). This means that the same cavity is formed by Ile1, Ile171 and Asp190. If we refer back to the temporal scatterplot in Figure 7-right, we notice that no cavity is present when we perform the query 176&193. However, this is not in agreement with the description of binding sites from the study by Hajjar et al. [34]. We postulate the following reason, for which we have not found a cavity represented by a single graph component, where Hajjar and coworkers see a single binding site. The cavity formed by both Ser176 and Val193, is likely to be composed of two distinct concave surface features that are divided by a surface extrusion. This hypothesis is supported by the amino acid query 176 ∨ 193, which shows that there are cavities formed by at least one of these amino acids (Figure 7). Such a compound cavity is not within the frame of our cavity description that requires the connectivity of the concave surface patch. Moreover, a difference between their and our study is that they analyze which part of PR3 interact with ligands (no cavity analysis) to derive a model of the binding sites, while we are looking at actual cavities on the molecular surface. That might explain the apparent discrepancy.

**Figure 9:**
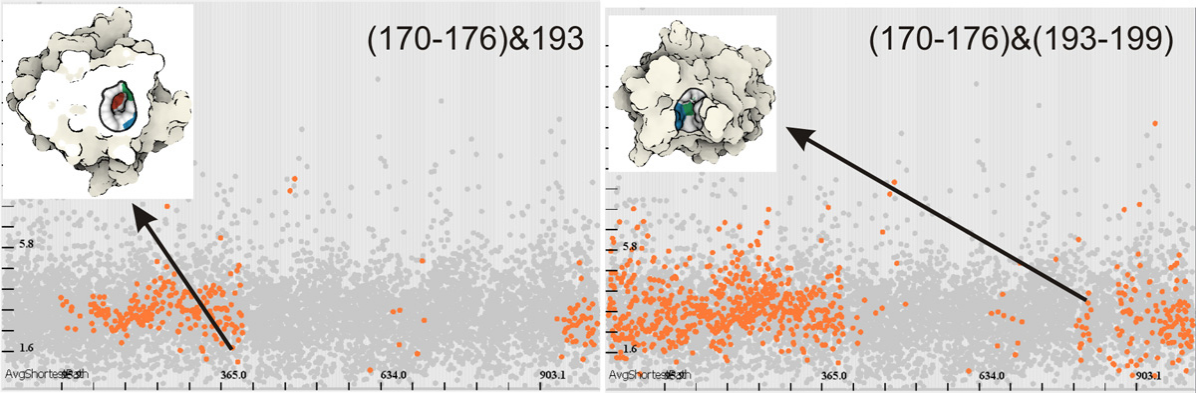
**Extending amino acids selection**. Left: We expand firstly the selection by 170 - 176&193, where we can see that the cavity appeared in the beginning and in the end of the simulation predominantly. Right: We expand selection furthermore by (170 - 176)&(193 - 199), where we notice that even more cavities appeared although still not as many in the middle of the simulation. We show a typical cavity for both of selections.

As a consequence, and as a next step, we perform several extended selections to see whether other amino acids might contribute to the cavity (Figure 9).

### Unsupervised cavity discovery

Hajjar et al. investigated the so-called *S*4 *− S*1 and *S*1' *− S*3' binding sites of Proteinase 3, and for doing so they performed analysis of numerous MD trajectories of PR3 with ligands. The design of their simulations and subsequent analyses were directed solely towards these binding sites and did not investigate other potential binding sites.

In the case of PR3, for which we analyzed the same MD simulation as was described in the previous section, we are able to discover cavities distinct from the known peptide binding sites; in particular one clear polar/hydrophobic but also with Arg (positively charged amino acid). By finding this cavity we have highlighted a region of the Proteinase 3 that has potentially an important role for its function. This cavity can be further characterized by our colleagues in molecular biology, who have the possibility to design experiments to investigate its potential functional role.

**Figure 10:**
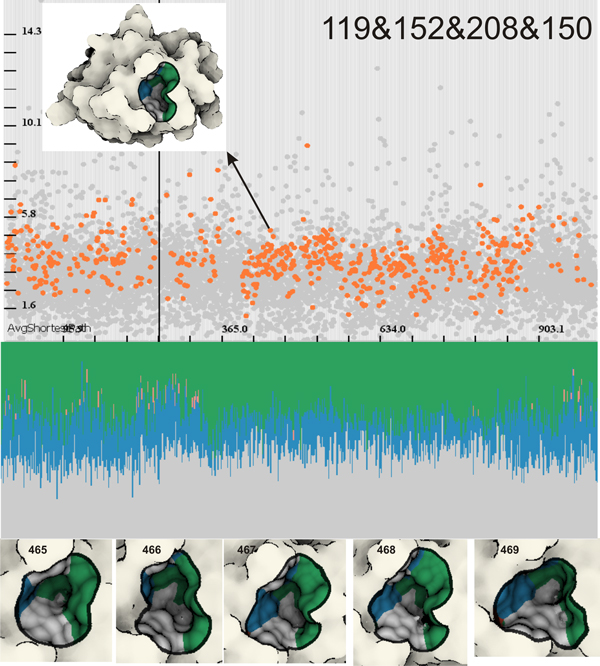
**A demonstration of a cavity formed by Val119, Val150, Thr152 and Arg208**. Top: In the temporal scatterplot we see that this cavity is frequently present over the entire temporal domain. Middle: The chemical properties are very stable as well, where the dominant ones are hydrophobicity and polarity. We can also note a small positively charged cavity characteristic. Bottom: A close-up on the cavity in five consecutive time steps (465,46,467,468 and 469). We can also observe the actual chemical properties directly in the vicinity of the cavity surface.

Since each graph component/cavity contains a list of participating amino acids, we can easily compute the most present amino acids over the entire simulation. By ordering the amino acids by their occurrence we made a list of the four most present amino acids, and we performed *AND *operations between all of them. These amino acids are: Val119, Val150, Thr152 and Arg208. We verify the presence of the graph components in the scatterplot (Figure 10 top), and the cavity shape and its span on the molecular surface in the 3*D *view (Figure 10 bottom). Then we can continue with the analysis of the chemical properties. Additionally, we estimated that the cavity graph formed by at least Val119 is present in the simulation for 86.5% of the total time, while all four amino acids form the cavity for 63% of the total time. We showcase this cavity in Figure 10, where we also see its chemical properties over time.

## Conclusions

We introduced a framework capable of detecting and visualizing cavities in molecular simulations. Furthermore the cavities are described by means of graphs, for which we compute graph attributes and a list of amino acids that constitute the molecular surface around the cavity graph. We used a brushing and linking methodology to analyze the graph attributes through dedicated views. We proposed a visualization method to show cavities in the context of the molecule. Additionally we introduced an implicit clipping plane that let us visually investigate occluded cavities localized inside the molecule.

Moreover, we have shown that our system enables to verify existing cavities through specification of amino acids of interest. We studied cavities defined by logical operators of the amino acids Ile1, Ile171, Ser176, Asp190, Ser191 and 193 in Proteinase 3 MD simulation. Additionally, we found out that there might be another cavity formed by at least four amino acids Val119, Thr152, Arg208 and Val150, which were even more persistent than the known ones. Our collaborators in biology agreed to study the discovered cavity more deeply.

One of the major limitations in our cavity extraction approach relates to the definition of the cavity. As already mentioned, the cavity is considered as a concave surface depression with a possible narrow opening when located on the molecular surface. To detect also shallow surface cavities, we can cast multiple rays from the sample point in distinct directions. However, such an approach will produce many false positives, which still can be reduced by the accompanied linking and brushing mechanism. This represents our future studies.

Another task that was demanded by our collaborators from biology was to track graph components over time. This is partly solved by linking amino acid selections. Nevertheless, it might happen that more than one cavity touches the same amino acid. This can be tackled by graph matching method applied on pair-wise graph components located in neighboring time steps. Here, possible scenarios of graph developments cover mainly splitting and merging of graph components between sequential time steps.

Another challenge is represented by incorporating charges into the existing concept. Electron potential charges are usually solved on the discrete volumetric grid by means of solving PDE. Since the implicit representation evaluates the function values anyway for any point in space, both representations can be easily merged. The resulting charges can then be mapped both to the graph components and to the iso-surface of the molecule.

## List of abbreviations used

vdW: van der Waals; SAS: Solvent Accessible Surface; SES: Solvent Excluded Surface; PR3: Proteinase 3; ICP: Implicit Clipping Plane; GLSL: OpenGL Shading Language; GPU: Graphics Processing Unit; CUDA: Compute Unified Device Architecture.

## Competing interests

The authors declare that they have no competing interests.

## Authors' contributions

JP developed the major framework and algorithms, implemented the 3*D *visualization and the 2*D *views related to amino acids. CT developed the 2D component view, implemented the brushing and linking technology, and the views for graphs components to enable the IVA process. IV brought focus and context visualization ideas. Additionally, he contributed to development of the cavity detection algorithm. NR provided the MD data, introduced the biological background and suggested to focus on amino acids, their detection algorithm, and discussed the cavity evaluation and discoveries. All authors wrote, read and approved the manuscript.

## Declarations

This publication was funded by the IEEE Symposium on Biological Data Visualization (BioVis) as a supplement of highlights.

The articles in this supplement have undergone the journal's standard peer review process for supplements. The Supplement Editors declare that they have no competing interests.

This article has been published as part of *BMC Bioinformatics *Volume 14 Supplement 19, 2013: Highlights from the 2nd IEEE Symposium on Biological Data Visualization. The full contents of the supplement are available online at http://www.biomedcentral.com/bmcbioinformatics/supplements/14/S19.
